# Innovative approaches to fall prevention in community‐dwelling older adults

**DOI:** 10.5694/mja2.52714

**Published:** 2025-06-24

**Authors:** Kim Delbaere, Catherine Sherrington, Catherine M Said, Vasikaran Naganathan

**Affiliations:** ^1^ Falls, Balance and Injury Research Centre Neuroscience Research Australia Sydney NSW; ^2^ University of New South Wales Sydney NSW; ^3^ Institute for Musculoskeletal Health, Sydney Local Health District Sydney NSW; ^4^ University of Sydney Sydney NSW; ^5^ University of Melbourne Melbourne VIC; ^6^ Western Health Melbourne VIC; ^7^ Centre for Education and Research on Ageing Sydney Local Health District Sydney NSW

**Keywords:** Falls, Accident prevention, Exercise therapy

Falls among community‐dwelling older adults represent a major public health challenge in Australia, and globally. Every day, about 400 older Australians are admitted to hospital due to falls. The consequences can be life‐changing, with fall‐related injuries often leading to long term disability, loss of independence, social isolation and premature entry into long term care. These impacts extend beyond the individual, placing significant strain on families, carers and the broader health care system. Falls cost more than $2.8 billion annually and reduce the availability of health care resources for people of all ages.^1^ Despite evidence from more than 600 trials — summarised across 12 Cochrane reviews — indicating that 20–30% of falls are preventable,^2^ fall prevention in Australia remains fragmented, underfunded and inconsistently implemented.^3^ This perspective article reviews the evidence on traditional and innovative approaches to fall prevention in community‐dwelling older adults, highlighting hybrid models that combine in‐person programs with digital solutions to improve accessibility and cost‐effectiveness. A summary of these traditional and innovative approaches is presented in the Box.

Box 1 **Summary of fall prevention approaches in community‐dwelling older adults**

**Table:** 

**Traditional approaches** *Note: Risk categories are based on the World guidelines for falls prevention and management for older adults* ^4^ *and reflect the likelihood of future falls*. Low risk: health promotion and community‐based group exercise Intermediate risk: tailored exercise and home safety interventions High risk: multifactorial assessments and multidisciplinary interventions (eg, tailored exercise, medication review, vision correction) Other: public health campaigns **Innovative approaches** *Note: Approaches can be tailored for use across risk levels depending on user needs, user preferences and clinical judgement*. Remote digital exercise programs (eg, StandingTall program, smart+step) Telehealth interventions (eg, TOP UP program) Simulation‐based balance training (eg, reactive balance, safe landing) Digital tools for caregiver training and medication management (eg, STOPPFall) Hybrid models combining digital access with periodic in‐person or phone‐based support

## Traditional approaches

The *World guidelines for falls prevention and management for older adults* recommend a stratified approach to fall prevention: (i) health promotion and community exercise for older adults at low risk; (ii) tailored exercise and home safety interventions for those at intermediate risk; and (iii) comprehensive multifactorial assessments and targeted medical interventions (eg, medication reviews, cataract surgery) for those at high risk.^4^ Multifactorial interventions, combining assessment with medical and allied health management, can be highly effective by addressing multiple risk factors simultaneously.^5^ However, these programs are resource‐intensive and logistically challenging, particularly for older adults in rural or remote areas, and long waiting lists limit timely and equitable access. Population‐based interventions, such as public health campaigns, have also been explored, but their effectiveness remains unclear,^6^ unless supported by funded, local services.^7^ Structured exercise programs remain the strongest evidence‐based solution. An updated Cochrane review of 116 trials demonstrated that exercise programs reduce fall rates by about 23%, with programs involving three hours per week and a focus on balance achieving reductions up to 42%.^2^ Home safety modifications and occupational therapy interventions can reduce falls by up to 38% among frailer, high risk groups.^8^ Community‐based fall prevention programs, such as Stepping On, the Otago Exercise Program and structured group exercise classes, have been successfully implemented beyond the clinical trial phase.^9^ These programs may also support social connection, providing mental health and wellbeing benefits beyond fall prevention. However, widespread adoption remains limited by short term funding, workforce shortages and fragmented collaboration across health care, community and aged care sectors. These challenges highlight the potential of digital and hybrid models to improve accessibility, scalability and sustainability.

## Innovative approaches

### Remote exercise programs

Several digital exercise programs have been shown to prevent falls in recent randomised clinical trials. For example, the smart+step “exergame” combining physical exercise with cognitive challenges in interactive, game‐like activities, reduced fall rates by 25% over 12 months.^10^ Similarly, the StandingTall program, delivering tailored balance and functional strength exercises via a tablet along with behavioural change strategies, reduced injurious fall rates by 20% in a 24‐month trial^11^ and was cost‐effective in older adults with previous falls.^12^ The TOP UP program (Tele‐guided Older People's program), a physiotherapy‐led intervention combining telehealth consultations with exercise delivery through video demonstrations, achieved high acceptance among community aged care clients and staff.^13^ Combining remote exercise programs with periodic in‐person sessions or telephone coaching^14^ could allow health care systems to provide personalised support based on real‐time monitoring. In addition, continuous activity monitoring through wearable sensors might further enable real‐time feedback and adjustments tailored to individual needs.^15^ An international implementation study confirmed higher‐than‐usual participant uptake and adherence to the StandingTall program in the community, yet it identified important barriers, including access to suitable devices and low digital literacy among some older adults.^16^ Other novel approaches, such as outdoor exercise parks designed for older adults, may also support engagement and functional mobility, although further evaluation is needed.^17^

### Simulation‐based balance training interventions

Emerging evidence suggests that incorporating simulations of real‐world challenges may improve the effectiveness of exercise programs. For example, simulation‐based balance training incorporating safe landing techniques, such as martial arts‐inspired tuck‐and‐roll or impact‐absorbing strategies, has potential to reduce impact forces and improve landing skills in older adults.^18^ Additionally, gait adaptability training (dynamic adaptation to obstacles),^19^ perturbation‐based training (responses to controlled perturbations),^20^ and immersive virtual reality (realistic environments engaging multiple sensory systems)^21^ have shown promise in enhancing balance and potentially reducing falls. However, larger randomised trials are needed to confirm their effectiveness and equipment requirements may limit the scalability of some approaches.^22^

### Caregiver training and medication management

Digital technologies are expanding beyond direct interventions for older adults. One innovative digital approach trains caregivers to conduct home safety assessments and implement low cost modifications to reduce environmental hazards in homes.^23^ Tools such as STOPPFall (Screening Tool of Older Persons Prescriptions in older adults with high fall risk) assist clinicians in systematically identifying and deprescribing medications and addressing critical issues of polypharmacy among older adults.^24^ Further research is needed to determine how best to implement these tools into routine practice at scale.

## Adoption of digital and hybrid models

Hybrid models, combining personalised face‐to‐face support with digital technologies, can extend the reach of fall prevention programs. Digital platforms delivering evidence‐based exercise with behavioural strategies can reach older adults unable to attend in‐person classes due to geographic isolation, mobility restrictions or limited local resources.^16^ Combining digital programs with periodic in‐person or phone‐based interactions promotes sustained engagement. For those unable to join group programs, online communities may also reduce social isolation. Successful adoption requires careful planning and adequate resourcing.^16^ Equitable access is critical, as older adults vary widely in their ability to access, use and benefit from technology. In particular, frail older adults require highly personalised care. Telehealth‐delivered exercise interventions show promising feasibility and acceptability among this group, although further research is needed to confirm long term effectiveness and optimal delivery methods.^25^ Additional barriers, such as limited English proficiency, lower education levels, low incomes or insufficient caregiver support, must be addressed through targeted strategies, including digital literacy training, culturally and linguistically tailored resources and affordable or subsidised access to technology and internet connectivity. Cross‐sector collaboration between health care providers, social services, community organisations and technology developers is essential to enable digital fall prevention solutions to become routine practice, supported by ongoing evaluation to confirm population‐level benefits.

## Future research directions

Despite the promise of digital and hybrid approaches, key evidence gaps remain. Future trials should explore tailored interventions for high risk groups, such as people living with dementia, osteoarthritis or Parkinson disease, where evidence is limited. Population‐level trials are needed to confirm efficacy, cost‐effectiveness and comparative value. Equally important is research on how best to implement these digital solutions into clinical workflows, particularly in the context of national aged care reforms. Ensuring digital interventions are equitable, accessible and acceptable for diverse populations is crucial. Digital literacy, cybersecurity and online safety are additional priorities, especially given concerns about scams targeting older adults. Research should identify ways to support older adults navigating digital environments safely, particularly those facing socio‐economic disadvantage, or with lower educational and limited caregiver support.

## Call to action

Australia's lack of a coordinated national fall prevention policy or strategy represents a critical gap in public health efforts, particularly given our rapidly ageing population and increasing fall‐related morbidity and mortality. If Australia is committed to reducing the significant burden of falls, we need to fundamentally change how we approach fall prevention. A national mass media campaign could help raise awareness of this neglected health problem, supported by a trusted online resource providing clear, evidence‐based information to older Australians, families and health professionals. However, increased public awareness alone is not enough. Older adults need better access to proven services such as balance exercise programs, home modifications and medical professionals trained in fall prevention.^4^ Recent digital solutions and hybrid models offer scalable, practical solutions capable of complementing traditional approaches.^16^ Evidence‐based digital programs are ready to be systematically implemented into health and aged care systems, supported by periodic in‐person or telehealth contact to improve access, reach and engagement. Traditional approaches must remain available, ensuring fall prevention remains inclusive and equitable for all older Australians. Stable, long term funding is essential to sustain a skilled workforce and provide continuous professional development for all involved in fall prevention. Policy makers should encourage system‐wide collaboration across health, aged care and community sectors (public and private), supported by sustainable funding and incentives for person‐centred care that actively involves older adults. Finally, national evaluation frameworks and data dashboards are needed to transparently track outcomes, adapt programs if needed and demonstrate a return on investment to both individuals and the broader health system.

## Conclusion

Fall prevention is a critical challenge, but also an opportunity. Guided by a coordinated national strategy that combines sustained investment in proven traditional approaches with the scalable potential of digital and hybrid models — along with equitable access, ongoing workforce development and system‐wide collaboration — Australia can reduce fall‐related injuries, disability, social isolation and premature entry into long term care. This will ultimately improve the health and quality of life for older Australians. Combined with national awareness campaigns, a centralised online resource and continuous evaluation to demonstrate impact and drive improvement, these efforts will deliver a sustainable approach to fall prevention.

## Open access

Open access publishing facilitated by University of New South Wales, as part of the Wiley ‐ University of New South Wales agreement via the Council of Australian University Librarians.

## Competing interests

Kim Delbaere is the scientific founder of a company established to commercialise the StandingTall program, which is mentioned in this article. At the time of publishing, there are no commercial interests or financial relationships in place.

## Provenance

Commissioned; externally peer reviewed.

## Author contributions

Delbaere K: Conceptualization, writing – original draft, writing – review and editing. Sherrington C: Writing – review and editing. Said C: Writing – review and editing. Naganathan V: Writing – review and editing.

## References

[1] Delbaere K , Elkington J , Lord SR , et al. The rising cost of falls – health researchers are calling for action. Australas J Ageing 2022; 41: 487‐489. 36458650 10.1111/ajag.13157

[2] Sherrington C , Fairhall N , Kwok W , et al. Evidence on physical activity and falls prevention for people aged 65+ years: systematic review to inform the WHO guidelines on physical activity and sedentary behaviour. Int J Behav Nutr Phys Act 2020; 17: 144. 33239019 10.1186/s12966-020-01041-3 PMC7689963

[3] Costa N , Ambrens M , Delbaere K , et al. A systems approach to assist policy action to prevent falls among community‐dwelling older people in Australia. Public Health Res Pract 2024; 34: 3412405. 38569571 10.17061/phrp3412405

[4] Montero‐Odasso M , van der Velde N , Martin FC , et al. World guidelines for falls prevention and management for older adults: a global initiative. Age Ageing 2022; 51: afac205. 36178003 10.1093/ageing/afac205 PMC9523684

[5] Lee SH , Yu S . Effectiveness of multifactorial interventions in preventing falls among older adults in the community: a systematic review and meta‐analysis. Int J Nurs Stud 2020; 106: 103564. 32272282 10.1016/j.ijnurstu.2020.103564

[6] Lewis SR , McGarrigle L , Pritchard MW , et al. Population‐based interventions for preventing falls and fall‐related injuries in older people. Cochrane Database Syst Rev 2024; 1: CD013789. 38180112 10.1002/14651858.CD013789.pub2 PMC10767771

[7] Baker PR , Francis DP , Soares J , et al. Community wide interventions for increasing physical activity. Cochrane Database Syst Rev 2015; 1: CD008366. 25556970 10.1002/14651858.CD008366.pub3 PMC9508615

[8] Clemson L , Stark S , Pighills AC , et al. Environmental interventions for preventing falls in older people living in the community. Cochrane Database Syst Rev 2023; 3: CD013258. 36893804 10.1002/14651858.CD013258.pub2 PMC9998238

[9] Paul SS , Li Q , Harvey L , et al. Scale‐up of the Stepping On fall prevention program amongst older adults in NSW: program reach and fall‐related health service use. Health Promot J Austr 2021; 32 (Suppl 2): 391‐398. 10.1002/hpja.413 32860442

[10] Sturnieks DL , Hicks C , Smith N , et al. Exergame and cognitive training for preventing falls in community‐dwelling older people: a randomized controlled trial. Nat Med 2024; 30: 98‐105. 38228913 10.1038/s41591-023-02739-0

[11] Delbaere K , Valenzuela T , Lord SR , et al. E‐health StandingTall balance exercise for fall prevention in older people: results of a two year randomised controlled trial. BMJ 2021; 373: n740. 33824131 10.1136/bmj.n740 PMC8022322

[12] Ambrens M , van Schooten KS , Lung T , et al. Economic evaluation of the e‐Health StandingTall balance exercise programme for fall prevention in people aged 70 years and over. Age Ageing 2022; 51: afac130. 35679193 10.1093/ageing/afac130

[13] Dawson R , Gilchrist H , Pinheiro M , et al. Experiences of older adults, physiotherapists, and aged care staff in the TOP UP Telephysiotherapy Program: interview study of the TOP UP interventions. JMIR Aging 2024; 7: e53010. 38324369 10.2196/53010 PMC10882472

[14] Oliveira JS , Sherrington C , Rissel C , et al. Effect of a coaching intervention to enhance physical activity and prevent falls in community‐dwelling people aged 60+ years: a cluster randomised controlled trial. Br J Sports Med 2024; 58: 382‐391. 38253435 10.1136/bjsports-2023-107027 PMC10982628

[15] Nouredanesh M , Godfrey A , Howcroft J , et al. Fall risk assessment in the wild: a critical examination of wearable sensor use in free‐living conditions. Gait Posture 2021; 85: 178‐190. 33601319 10.1016/j.gaitpost.2020.04.010

[16] Taylor ME , Ambrens M , Hawley‐Hague H , et al. Implementation of a digital exercise programme in health services to prevent falls in older people. Age Ageing 2024; 53: afae173. 39113467 10.1093/ageing/afae173 PMC11306314

[17] Levinger P , Dunn J , Panisset MG , et al. The effect of the ENJOY Seniors Exercise Park Physical Activity Program on falls in older people in the community: a prospective pre‐post study design. J Nutr Health Aging 2022; 26: 217‐221. 35297462 10.1007/s12603-021-1724-1 PMC8727466

[18] Moon Y , Sosnoff JJ . Safe landing strategies during a fall: systematic review and meta‐analysis. Arch Phys Med Rehabil 2017; 98: 783‐794. 27592402 10.1016/j.apmr.2016.08.460

[19] Nørgaard JE , Jorgensen MG , Ryg J , et al. Effects of gait adaptability training on falls and fall‐related fractures in older adults: a systematic review and meta‐analysis. Age Ageing 2021; 50: 1914‐1924. 34120163 10.1093/ageing/afab105

[20] Devasahayam AJ , Farwell K , Lim B , et al. The effect of reactive balance training on falls in daily life: an updated systematic review and meta‐analysis. Phys Ther 2023; 103: pzac154. 10.1093/ptj/pzac154 37651698

[21] Lee J , Phu S , Lord SR , Okubo Y . Effects of immersive virtual reality training on balance, gait and mobility in older adults: a systematic review and meta‐analysis. Gait Posture 2024; 110: 129‐137. 38581933 10.1016/j.gaitpost.2024.03.009

[22] McCrum C , Bhatt TS , Gerards MHG , et al. Perturbation‐based balance training: principles, mechanisms and implementation in clinical practice. Front Sports Act Living 2022; 4: 1015394. 36275443 10.3389/fspor.2022.1015394 PMC9583884

[23] Dehnavi M , Valizadeh Zare N , Mazlom SR , et al. Investigating the effect of using a home safety training application by caregivers on accident risk management in the elderly. Exp Gerontol 2025; 199: 112661. 39694449 10.1016/j.exger.2024.112661

[24] Seppala LJ , Petrovic M , Ryg J , et al. STOPPFall (Screening Tool of Older Persons Prescriptions in older adults with high fall risk): a Delphi study by the EuGMS Task and Finish Group on fall‐risk‐increasing drugs. Age Ageing 2021; 50: 1189‐1199. 33349863 10.1093/ageing/afaa249 PMC8244563

[25] Dawson R , Oliveira JS , Kwok WS , et al. Exercise interventions delivered through telehealth to improve physical functioning for older adults with frailty, cognitive, or mobility disability: a systematic review and meta‐analysis. Telemed J E Health 2024; 30: 940‐950. 37975811 10.1089/tmj.2023.0177 PMC11035924

